# Selective Glucocorticoid Receptor Properties of GSK866 Analogs with Cysteine Reactive Warheads

**DOI:** 10.3389/fimmu.2017.01324

**Published:** 2017-11-01

**Authors:** Chandra S. Chirumamilla, Ajay Palagani, Balu Kamaraj, Ken Declerck, Marinus W. C. Verbeek, Ryabtsova Oksana, Karolien De Bosscher, Nadia Bougarne, Bart Ruttens, Kris Gevaert, René Houtman, Winnok H. De Vos, Jurgen Joossens, Pieter Van Der Veken, Koen Augustyns, Xaveer Van Ostade, Annemie Bogaerts, Hans De Winter, Wim Vanden Berghe

**Affiliations:** ^1^Laboratory of Protein Chemistry, Proteomics and Epigenetic Signalling, Department of Biomedical Sciences, University of Antwerp, Antwerp, Belgium; ^2^Research Group PLASMANT, Department of Chemistry, University of Antwerp, Antwerp, Belgium; ^3^Medicinal Chemistry, Department of Pharmaceutical Sciences, University of Antwerp, Antwerp, Belgium; ^4^Receptor Research Laboratories, Nuclear Receptor Lab (NRL) and Cytokine Receptor Lab (CRL), VIB-UGent Center for Medical Biotechnology, Ghent University, Ghent, Belgium; ^5^Center for Medical Biotechnology, Department of Biochemistry, VIB, Ghent University, Ghent, Belgium; ^6^PamGene International B.V., Den Bosch, Netherlands; ^7^Laboratory of Cell Biology and Histology, Department of Veterinary Sciences, University of Antwerp, Antwerp, Belgium

**Keywords:** glucocorticoid receptor, SEGRA, NFkB, electrophilic, covalent warhead, cysteine

## Abstract

Synthetic glucocorticoids (GC) are the mainstay therapy for treatment of acute and chronic inflammatory disorders. Due to the high adverse effects associated with long-term use, GC pharmacology has focused since the nineties on more selective GC ligand-binding strategies, classified as selective glucocorticoid receptor (GR) agonists (SEGRAs) or selective glucocorticoid receptor modulators (SEGRMs). In the current study, GSK866 analogs with electrophilic covalent-binding warheads were developed with potential SEGRA properties to improve their clinical safety profile for long-lasting topical skin disease applications. Since the off-rate of a covalently binding drug is negligible compared to that of a non-covalent drug, its therapeutic effects can be prolonged and typically, smaller doses of the drug are necessary to reach the same level of therapeutic efficacy, thereby potentially reducing systemic side effects. Different analogs of SEGRA GSK866 coupled to cysteine reactive warheads were characterized for GR potency and selectivity in various biochemical and cellular assays. GR- and NFκB-dependent reporter gene studies show favorable anti-inflammatory properties with reduced GR transactivation of two non-steroidal GSK866 analogs UAMC-1217 and UAMC-1218, whereas UAMC-1158 and UAMC-1159 compounds failed to modulate cellular GR activity. These results were further supported by GR immuno-localization and S211 phospho-GR western analysis, illustrating significant GR phosphoactivation and nuclear translocation upon treatment of GSK866, UAMC-1217, or UAMC-1218, but not in case of UAMC-1158 or UAMC-1159. Furthermore, mass spectrometry analysis of tryptic peptides of recombinant GR ligand-binding domain (LBD) bound to UAMC-1217 or UAMC-1218 confirmed covalent cysteine-dependent GR binding. Finally, molecular dynamics simulations, as well as glucocorticoid receptor ligand-binding domain (GR-LBD) coregulator interaction profiling of the GR-LBD bound to GSK866 or its covalently binding analogs UAMC-1217 or UAMC-1218 revealed subtle conformational differences that might underlie their SEGRA properties. Altogether, GSK866 analogs UAMC-1217 and UAMC-1218 hold promise as a novel class of covalent-binding SEGRA ligands for the treatment of topical inflammatory skin disorders.

## Introduction

Synthetic glucocorticoids (GCs) such as prednisolone, dexamethasone, or fluticasone esters remain the frontline treatment for (chronic) inflammatory disorders, autoimmune diseases, and/or hematological malignancies (1). Therapeutic activities of synthetic GCs are mediated by the glucocorticoid hormone receptor (GR) belonging to a superfamily of ligand-inducible transcription factors. In the absence of GCs, the GR resides in the cytosol in an inactive state complexed with heat shock proteins (HSPs) and immunophilins (2). Binding of GCs to the GR activates and then translocates to the nucleus by causing a conformational change in the GR followed by a dissociation of the bound HSPs. The activated GR can then regulate gene activation by GR dimerization (transactivation) and binding to glucocorticoid response elements (GREs) in the genome, or regulate gene repression by GR monomers (transrepression) which interfere with the activity of pro-inflammatory transcription factors, such as NFκB and AP1 (3, 4). Unfortunately, major metabolic side effects, such as glucose intolerance, muscle wasting, skin thinning (atrophy), and osteoporosis, severely limit their prolonged therapeutic use (5). Studies in animal models have shown that the GC-mediated side effects, such as diabetogenic activity, osteoporosis, and skin atrophy, are mainly due to the activation of the transactivation pathway (6, 7). Hence, a selective GC that is able to repress inflammation without transactivation should preserve many of the desirable therapeutic anti-inflammatory effects, while minimizing undesired side effects (8–11). This was also demonstrated by the introducing a point mutation in the GR^dim^ mice that prevented GR from dimerizing and binding to DNA, thereby blocking transactivation without interfering with anti-inflammatory transrepression effects (12). Therefore, new GC pharmacological approaches are being developed that attempt to amplify the therapeutic beneficial anti-inflammatory (transrepression) actions and to minimize adverse metabolic (transactivation) activities. These compounds are classified as selective glucocorticoid receptor agonists (SEGRAs) or selective glucocorticoid receptor modulators (SEGRMs) (7, 13, 14). Various crystal structures of nuclear hormone receptor binding domains co-crystallized with different ligands demonstrate subtle conformational changes of the GR ligand-binding domain (LBD) which result in variable agonistic/antagonistic or mixed pharmacological properties depending on specific coactivator or corepressor interactions (14–17). Since the late 1990s, this has triggered researchers to synthetize various SEGRA and SEGRM compounds, with GSK866 and mapracorat being the prototypes of this non-steroidal class of compounds, in which bulky, bicyclic aromatic substituents account for the structural similarity to corticoids (7, 13–15, 18–20). *In vivo* efficacy for SEGRA/SEGRM compounds with reduced side effects has already been demonstrated for treatment of acute infections, such as rheumatoid arthritis, asthma, and colitis, in the clinic (20–22).

In the current study, GSK866 analogs with electrophilic warheads were evaluated for potential SEGRA properties to improve their clinical safety profile for long-lasting topical skin disease applications (14). Of particular interest, reduced GR transactivation was observed for GR, covalently bound to dexamethasone-21-mesylate (23, 24). This proof-of-concept study builds further upon the ideas that emerged from the recent development of covalent-binding kinase inhibitors (25–28), with the underlying idea to develop long-lasting GR agonists with applicability in the anti-inflammatory domain. Furthermore, evidence suggests that there is a reduced risk for the development of resistance against covalent drugs, which is a major challenge in areas such as oncology and infectious disease (28, 29). When drugs bind their target covalently, the off-rate is negligible compared to that of a non-covalent drug and, therefore, such covalently binding drugs should have a prolonged therapeutic effect *in vivo*. Another advantage of the irreversible binding is that smaller doses of the drug are necessary to obtain therapeutic efficacy and systemic side effects can be reduced. Covalent cysteine binding drugs have gained renewed interest, since the Food and Drug Administration (FDA) awarded a “breakthrough drug” status to cysteine-directed covalent inhibitors that target Bruton’s tyrosine kinase (BTK) and a drug-resistant EGFR mutant in cancer (26, 28–30). In addition, most widely prescribed cancer drugs, such as the proteasome inhibitor bortezomib, CYP17 inhibitor abiraterone acetate and P6H5 enzyme (Cyclooxygenase) inhibitor widely known as aspirin, were also found to bind their targets covalently (31–33). However, possible therapeutic applications for potential covalent-binding SEGRAs remain to be explored.

In the current study, we performed structure function analysis of different GSK866 analogs with cysteine reactive warheads to evaluate potential SEGRA properties by different biochemical and cellular assays, including (reporter) gene expression analysis, immuno-localization studies, proteomic GR binding studies, glucocorticoid receptor (GR)-coregulator interaction profiling, and molecular dynamic simulation.

## Materials and Methods

### Cell Culture and Materials

TNF-sensitive L929sA fibrosarcoma cells have previously been characterized in our lab (34–36). A549 adenocarcinoma human alveolar basal epithelial cells were a kind gift from Dr. Ian Adcock and have previously been described (35–37). Fibrosarcoma and adenocarcinoma cells were grown in Dulbecco’s modified Eagle medium (DMEM, Invitrogen) with 10% fetal calf serum (FCS, PAA) 100 U/ml penicillin and 100 mg/ml streptomycin (PAA). Stable transfected NFκB-luc and GRE-luc reporter gene cell lines have been described previously (8, 38). HaCat keratinocyte cells were purchased from the ATCC (American Type Culture Collection, USA) and kept in DMEM high glucose (Invitrogen) with 10% FCS, 100 U/ml penicillin, and 100 mg/ml streptomycin. Typically, cells were seeded at a density of 30,000 cells/cm^2^ 1 day before the experiment. Synthesis of Dex-mesylate (Dex-Mes) and SEGRA GSK866 analogs UAMC-1158, -1159, -1217, and -1218 has been previously described (14). Dexamethasone and RU486 were purchased from SIGMA-Aldrich. All compounds were routinely dissolved in DMSO at a concentration of 10^–2^ M. For cell culture and other experiments, compounds were further diluted to their final concentration in DMEM media without antibiotics and serum. For experiments, the cells were seeded on multi-well dishes and allowed to attach for 24 h. Prior to treatment with University of Antwerp Medicinal Chemistry (UAMC) compounds or Dex stimulation, cells were cultured in low FCS (<2%) for about 12 h.

### Immunolocalization Studies

To detect endogenous GR in A549 cells, cells were grown in serum free media for 24 h followed by treatment with 1 µM of specific GR ligands. After 30 min of treatment, cells on the cover glass were fixed in 4% formaldehyde in phosphate-buffered saline (PBS), permeabilized with 0.3% Triton X-100–PBS, and then blocked in 5% milk, 10% FCS, 0.3% bovine serum albumin, and 0.3% Triton X-100 in PBS for 30 min at room temperature. Primary antibodies against anti GR-antibody (SantaCruz Biotechnology) were used at a dilution of 1:200. Nuclei were stained by DAPI and samples were analyzed using a confocal Leica LSM410 microscope. To quantify nuclear translocation of GR in a standardized manner, multichannel fluorescence images were analyzed using a script for FIJI image analysis freeware (http://fiji.sc) that is essentially based on a pipeline described before (CellBlocks.ijm) (39), and is available upon request. In brief, the analysis first segments nuclei in the Hoechst/DAPI channel and then uses the nuclear mask to define perinuclear cytoplasmic regions of interest (ROIs). Nuclei are detected after local contrast enhancement and Gaussian smoothing to, respectively, cover for spatial illumination heterogeneity and noise. After automatic thresholding (Isodata algorithm), touching nuclei are separated using a conditional watershed algorithm (40). Next, cytoplasmic ROIs are determined as concentric bands around the nuclei by Voronoi-restrained region growing (41). Finally, the average intensity of the GR channel is measured in both the nuclear and cytoplasmic ROIs and the nuclear-to cytoplasm signal intensity ratio (N/C Ratio) is calculated per cell (Figure S1 in Supplementary Material) (42).

### Cell-Based Reporter Assays

In order to compare transactivation and transrepression potencies of the different synthesized GSK866 analogs, we performed GRE- and NFκB-luciferase-dependent reporter gene studies in stable transfected cell lines, as previously described (3, 8). HaCat cells expressing p(GRE)_2_ 50-Luc and L929sA cells expressing p(GRE)_2_50-Luc or p(NFκB)_3_50-Luc were cultured in DMEM medium with 1% l-glutamine, supplemented with 10% feral bovine serum (FBS), 100 µg/ml streptomycin, and 100 IU/mL penicillin in a humidified 5% CO_2_ atmosphere. Cells were passaged every 2 days at a dilution of 1:6. Reporter gene cells were plated at a density of 0.5 × 10^5^ cells/well in 24 well plates and grown overnight. p(GRE)_2_50-Luc-dependent reporter gene cells were treated with solvent (DMSO) or compound (100 fM, 1 nM, 10 nM, 100 nM, and 1 µM) for 6 h, whereas in case of p(NFκB)_3_50-Luc, TNF (2,500 IU/mL) was added 2 h following compound treatment. After incubation with the compounds, medium was removed and cells were washed twice with PBS followed by adding 100 µl of 1× lysis buffer (0.2 M K_2_HPO_4_, 0.2 M KH_2_PO_4_, Triton X-100, pH 7.6). 25 µl of lysate was assayed for luciferase activity by adding 50 µl of luciferase assay substrate (1 mM luciferin or luciferin salt, 3 mM ATP, and 15 mM MgSO_4_ in 30 mM HEPES buffer, pH 7.8) followed by 10 s mixing and measuring 1 s bioluminescence using an Envision multilabel reader (Perkin Elmer).

### qPCR Analysis

qPCR was performed using the GoTaq qPCR Master Mix (Promega) according to manufacturer’s instructions. In short, a 25 µl reaction volume mix per sample was prepared containing 12.5 µl GoTaq qPCR Master Mix, 0.4 µM forward and reverse primer, and nuclease-free water. We used the Rotor-Gene Q qPCR machine of Qiagen with following PCR program: 95°C for 2 min, 40 cycli denaturation (95°C, 15 s) and annealing/extension (60°C, 30 s), and dissociation (60–95°C). Following primers were used: GAPDH FP (housekeeping gene HKG) GCTCTCTGCTCCTCCTGTTC; GAPDH RP (HKG) ACGACCAAATCCGTTGACTC; IL6 FP GACAGCCACTCACCTCTTCA; IL6 RP AGTGCCTCTTTGCTGCTTTC; GILZ FP TCAGACAGGACTGGAACTTCTCC; GILZ RP GCGTGAGAACACCCTGTTGA. Each sample was run in triplicate. The median value of the triplicates were taken to calculate the deltaCt-values using GAPDH as the normalization gene.

### Western Blot Analysis

Whole cell extracts contain multiple protease inhibitors (Complete Mini^®^, Roche) and Halt phosphatase inhibitor cocktail (Thermoscientific). Soluble protein extracts were obtained after three times freeze and thaw cycles and centrifugation at 16 *g* for 15 min at 4°C. Samples were separated by sodium dodecyl sulfate-polyacrylamidegel electrophoresis (SDS-PAGE) and transferred onto nitrocellulose membranes (Hybond C, Amersham) following standard protocols. After blocking, membranes were incubated overnight at 4°C with the primary antibodies: anti-GR (sc-8992, SantaCruz Biotechnology), anti-phospho-GR Ser211 (4161, Cell Signalling) and anti-Actin (A3853, Sigma-Aldrich) followed by dye-conjugated secondary antibodies (LI-COR^®^ Biosciences). Bound complexes were detected with the Odyssey^®^ infrared imaging system (LI-COR^®^) and quantified by Image J software.

### Mass Spectrometry Analysis

Recombinant His6-MBP-mCaspase3 site-LBD-hGR was expressed in the *Escherichia coli* strain BL21codon + pICA2 that was transformed with the corresponding pLHM36LBD-hGR plasmid and in which expression was induced by Isopropyl β-D-1-thiogalactopyranoside (IPTG) under control of a pL-promotor. The transformed bacteria were grown in Luria Bertani medium supplemented with ampicillin (100 µg/ml) and kanamycin (50 µg/ml) overnight at 28°C before 1/100 inoculation in 12 × 0.5 l provided with Luria Bertani medium supplemented with ampicillin (100 µg/ml) and 1% glycerol in a shaker incubator. The cells were grown to an optical density at AU 600 nm of 0.65, transferred to 20°C and expression was induced by addition of 1 mM IPTG overnight. Cells were then harvested and frozen at −20°C. The cell pellet after production was 60 g. After thawing, the cells were re-suspended at 3 ml/g in cold 20 mM Tris-HCl pH 7.4, 200 mM NaCl, 1 mM EDTA, 1 tablet complete protease inhibitor cocktail from Roche/50 ml, and 10 mM β-mercaptoethanol. The cytoplasmic fraction was prepared by sonication of the cells on ice and isolated by centrifugation at 18,000 × *g* for 30 min. All further purification steps were conducted at 4°C. The clear supernatant was applied to an amylase column (BioLabs, XK26 × 20, 100 ml), equilibrated with 20 mM Tris–HCl pH 7.4, 200 mM NaCl, 1 mM EDTA, and 10 mM β-mercaptoethanol. The column was eluted with 20 mM Tris–HCl pH 7.4, 200 mM NaCl, 1 mM EDTA, 10 mM maltose, and 10 mM β-mercaptoethanol. The elution fractions were analyzed on SDS-PAGE with Coomassie staining and western blot with anti-His6 detection (data not shown). Recombinant purified His-MBP-DEVD-GR-LBD fusion protein was dissolved at a concentration of 1 mg in 1.7 ml PBS buffer. To 128 µl of His-MBP-DEVD-LBD stock solution (75 µg, 1.05 nmol), 3 µl (30 nmol) of Dex, Dex-Mes, UAMC-1158, UAMC-1159, GSK866, UAMC-1217, or UAMC-1218 stock solution (10 mM) or solvent (DMSO) was added.

Each aliquot was incubated 2 h at 37°C. Pierce Protein Desalting Spin Columns (max loading volume 120 µl) were equilibrated with 4 × 400 µl 20 mM NH_4_HCO_3_ (pH 7.8) (15.8 mg/10 ml) (1,500 × *g*, 1 min). 120 µl of each sample was loaded onto a spin column and the flow-through was collected (1,500 × *g*, 2 min). Trypsin (5 µl of 20 mM stock solution) was added, and the samples were incubated 20 h at 37°C. Then, 0.1% FA (formic acid) (41.5 µl) and 20 mM TCEP in 0.1% FA (18.5 µl, 5.7 mg TCEP/ml) were added [final volume = 185 µl, final concentration TCEP = 2 mM, 1 µg material/2.5 μl], and the samples were subjected to LC–MS/MS analysis.

The obtained peptide mixtures were introduced onto an LC–MS/MS system; the Ultimate 3000 RSLC nano (Dionex, Amsterdam, The Netherlands) in-line connected to an LTQ-Orbitrap Velos (Thermo Fisher Scientific, Bremen, Germany). The sample mixture was loaded on a trapping column [made in-house, 100 µm internal diameter (I.D.) × 20 mm, 5 µm C18 beads (Reprosil-HD, Dr. Maisch)]. After back flushing from the trapping column, the sample was loaded on a reverse-phase column [made in-house, 75 µm I.D. × 150 mm, 5 µm C18 beads (Reprosil-HD, Dr. Maisch)]. Peptides were loaded with solvent 1 (0.1% trifluoroacetic acid, 2% acetonitrile) and were separated with a linear gradient from 2% solvent 1’ (0.1% formic acid) to 55% solvent 2’ (0.1% formic acid and 80% acetonitrile) at a flow rate of 300 nl/min followed by a wash reaching 100% solvent 2’.

The mass spectrometer was operated in data-dependent mode, automatically switching between MS and MS/MS acquisition for the 10 most abundant peaks in a given MS spectrum. In the LTQ-Orbitrap Velos, full scan MS spectra were acquired in the Orbitrap at a target value of 1E6 with a resolution of 00060000. The 10 most intense ions were then isolated for fragmentation in the linear ion trap, with a dynamic exclusion of 30 s. Peptides were fragmented in HCD mode after filling the ion trap at a target value of 1E4 ion counts. From the MS/MS data in each LC run Mascot Generic Files were created using the Distiller software (version 2.4.3.3, Matrix Science, www.matrixscience.com/distiller.html). While generating these peak lists, grouping of spectra was allowed in Distiller with a maximum intermediate retention time of 30 s and a maximum intermediate scan count of 5 was used where possible. Grouping was done with 0.005 Th precursor tolerances. A peak list was only generated when the MS/MS spectrum contained more than 10 peaks. There was no de-isotoping and the relative signal to noise limit was set at 2. These peak lists were then searched with the Mascot search engine [MatrixScience, http://www.matrixscience.com (43)] using the Mascot Daemon interface (version 2.4.0, Matrix Science). Spectra were searched against the Swiss-Prot database (version 2012_11 of UniProtKB/Swiss-Prot protein database containing 538585 sequence entries of *homo sapiens*). Modifications of cysteine residues by GSK866, UAMC-1217, -1218 or Dex, or Des-Mes were included in the respective searches as an additional variable. For the reference experiment with DMSO, the same search parameters were used as for the other test compounds. Mass tolerance on peptide precursor ions was set to 10 ppm (with Mascot’s C13 option set to 1), and on fragment ions to 0.5 Da. The peptide charge was set to 1+, 2+, 3+ and instrument setting was put on ESI-TRAP. Enzyme was set to trypsin, allowing for one missed cleavage, also cleavage was allowed when arginine or lysine is followed by proline. Only peptides that were ranked one and scored above the threshold score, set at 99% confidence were withheld. Spectra identified as peptides modified with GSK866, UAMC-1158, -1159, -1217, -1218, or Dex were manually validated to ensure correct assignment. All data management was done by ms_lims (44).

### GR–Coregulator Interaction Profiling

Cofactor Interactions (coactivators and co-repressors) of recombinant glucocorticoid receptor ligand-binding domain (GR-LBD) complexed with different ligands Dex, GSK866, UAMC-1217, or UAMC-1218 were assessed by the Microarray Assay for Real-time Coregulator-Nuclear real-time coregulator-nuclear receptor Interaction (MARCoNI) as previously described (PamGene International B.V.) (16). In brief, all peptide array assays were performed on a Pamstation-12 at 20°C using two cycles per minute with 25 µl reaction mixture incubated for 40 min with Nuclear receptor buffer, 1 nM glutathione S-transferase (GST)-tagged GR-LBD (A15668, Life Technologies, USA), 25 nM, Alexa Fluor 488-conjugated GST antibody (A-11131), 1 µl DTT (0.05 mM), and MQ water or vehicle (2% DMSO in MQ water) with or without 1 μM receptor ligands Dex, GSK866, UAMC-1217 or UAMC-1218 or solvent control (2% DMSO in water). Each pamchip array was blocked with 30 µl of blocking buffer (1% BSA/1× TBS, 0.01% Tween 20). Next, the reaction-ligand mixture was incubated for 80 cycles. To analyze the results of the MARCoNI assay, we used the Bionavigator image analysis software (Version 5.1, Pamgene International B.V.). The software analyzes the automatically identified spots and quantifies intensities by calculating signal-minus background values and the receptor modulation index (MI) was subsequently calculated as the intensity of fluorescence change in presence of ligand over solvent control DMSO. All the images were obtained at cycles 21, 41, 61, and 81, respectively, by a CCD camera-based optical system integrated in the PamStation-96 instrument (PamGene International B.V.). The statistical significance of ligand effects on GR-LBD were calculated by using a Student’s *t*-test by an inbuilt Bionavigator application.

### Molecular Modeling

For covalent docking simulations in the GSK866 complexed GR-LBD (3E7C) structure, semi-flexible docking protocols were applied in Autodock Vina in which the binding residues of the target protein were kept flexible while the others were kept rigid (45). The Autodock Tools (GUI) program was used to add hydrogen atoms to the proteins and to merge non-polar contacts. Gasteiger charges were computed for the proteins. The grid box size was set at 20, 14, and 20 Å (*x, y*, and *z*), respectively. We optimized the total ligand–receptor interaction energy (ΔG) in kilocalorie per mole and its components by means of atomic level molecular mechanics simulations, based on a force field involving intermolecular van der Waals, electrostatic, and hydrogen bond interactions between the binding molecule and its receptor. The docked bioactive conformation graphs of the different ligands were captured by the PYMOL program (version 1.5.04, Schrodinger, LLC). Next cooperative bonds were characterized which may stabilize ligand receptor binding of the non-steroidal ligand GSK866.

### Molecular Dynamics Simulation

The GSK866 complexed form of the GR-LBD (3E7C) was subjected to molecular dynamics (MD) simulations using the GROMACS 4.5.5 tool (46). The GROMOS96 43A1 force field was used to prepare the protein topology file. The topological structures of GR ligands UAMC-1217 and Dex were generated by the PRODRG server (47). The systems were solvated with simple point charge (SPC) water molecules in a cubic box with dimensions 1 nm × 1 nm × 1 nm. Periodic boundary conditions were applied in all three (*x, y*, and *z*) directions to mimic the infinity of the system. At physiological pH conditions, the structures were found to be negatively charged. Hence, we added sodium ions (Na^+^) to make the system electrically neutral in the simulation box. The Berendsen temperature coupling method was applied to regulate the temperature inside the box. The particle mesh Ewald (PME) method (48) was used to treat the long-range electrostatic interactions. The pressure was maintained at 1 atm with an allowed compressibility range of 10^−5^ atm. The LINCS algorithm was used to constrain the bond lengths involving hydrogens, permitting a time step of 2 fs. Van der Waals and Coulomb interactions were truncated at 1.0 nm. The non-bonded pair list was updated every 10 steps and conformations were stored every 0.5 ps. Finally, the systems were subjected to MD simulations for 100 ns each at 300 K.

## Results

### Molecular Modeling of Cysteine Reactive GSK866 Analogs UAMC-1158, UAMC-1159, UAMC-1217, and UAMC-1218

**Figure 1:**
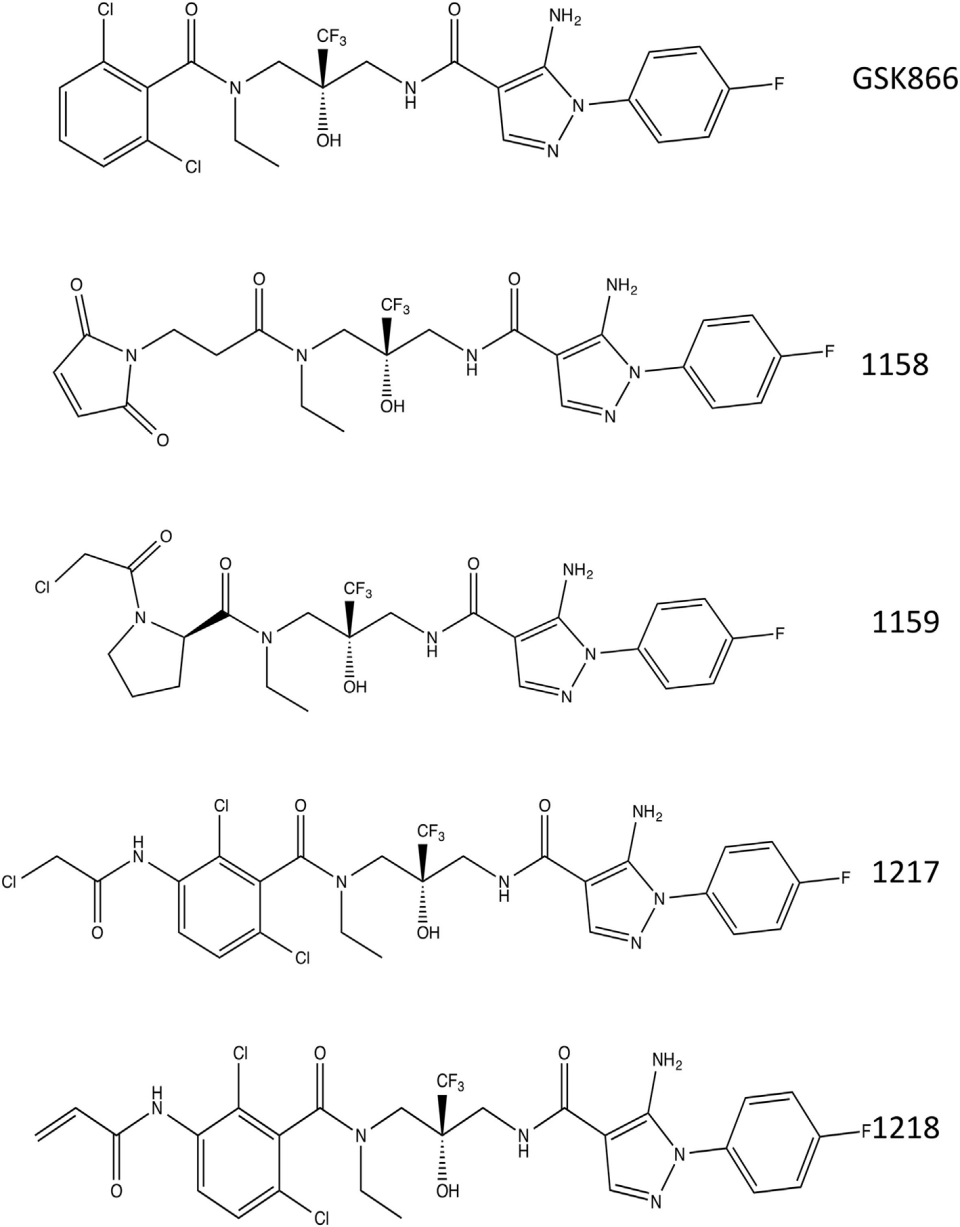
Chemical structures. Chemical structures of selective glucocorticoid receptor agonist GSK866 and synthetic analogs UAMC-1158, UAMC-1159, UAMC-1217, and UAMC-1218.

**Figure 2:**
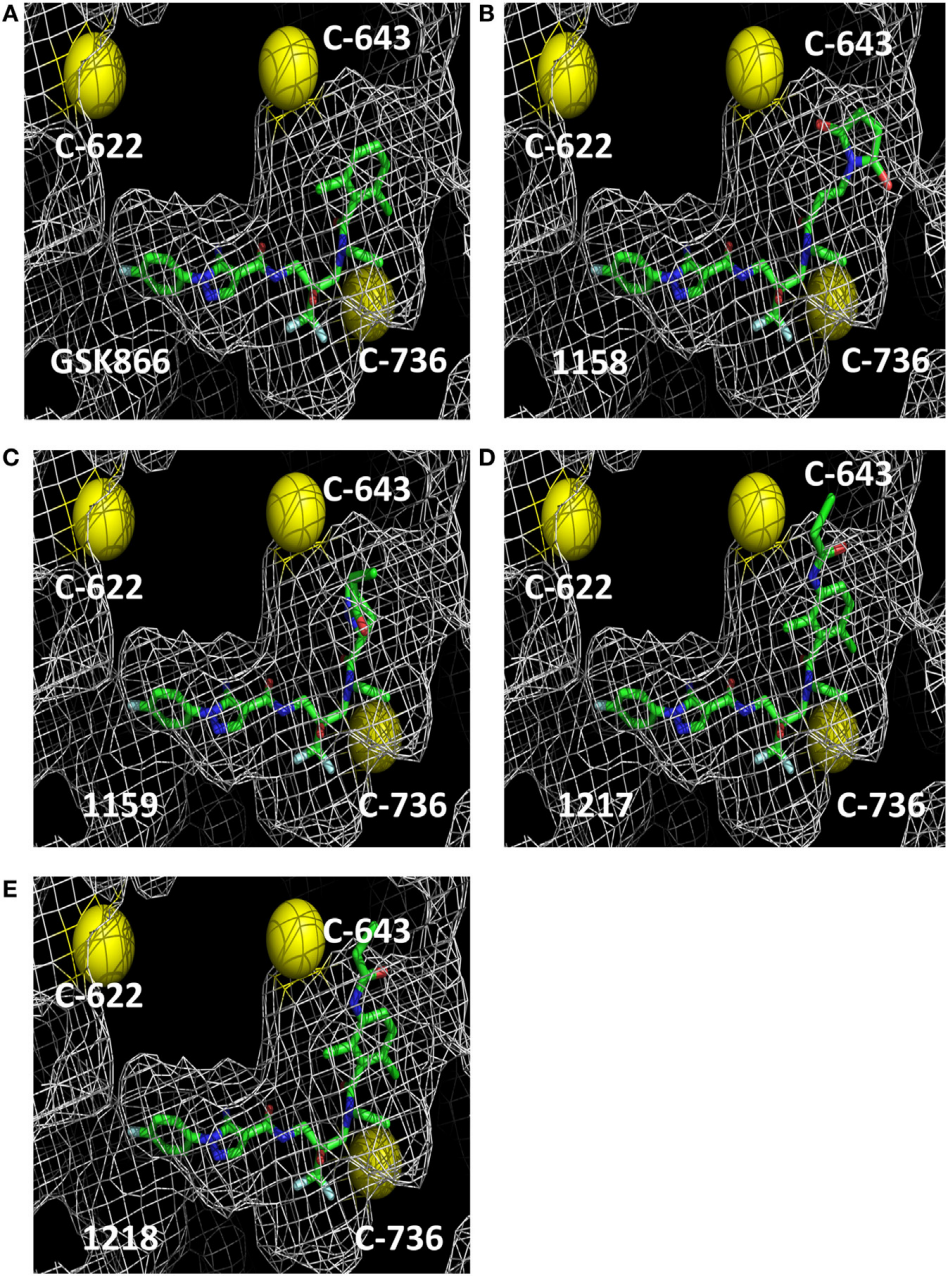
Molecular 3D modeling of cysteine reactive GSK866 analogs UAMC-1158, UAMC-1159, UAMC-1217, and UAMC-1218. Molecular 3D modeling of SEGRA GSK866 **(A)** and synthetic analogs UAMC-1158 **(B)**, UAMC-1159 **(C)**, UAMC-1217 **(D)**, and UAMC-1218 **(E)** in the glucocorticoid receptor ligand-binding domain crystal structure 3E7C. Proximal cysteine residues C622, C643, and C736 are represented as yellow balls.

The crystal structure of the ligand-binding domain of the GR has been solved in complex with SEGRA compound GSK866 (Figure 1), and this structure (pdb-code 3E7C) shows the location of a GR-LBD cysteine residue (C643) in close proximity to the dichlorophenyl group of SEGRA compound GSK866 (Figure 2A). Yellow spheres highlight the three-cysteine residues that are located in the vicinity of the ligand-binding pocket. The close presence of such a cysteine residue prompted us to design and synthesize different GSK866 analogs UAMC-1158, UAMC-1159, UAMC-1217, and UAMC-1218 (Figure 1) by introducing a reactive warhead into the structure, with the possibility of forming a covalent linkage between protein structure and ligand, upon binding (Figures 2B–E) (14).

### SEGRA GSK866 Analogs UAMC-1217, UAMC-1218 but Not UAMC-1158 and UAMC-1159 Compounds Elicit Selective GR-Transactivation and NFκB-Transrepression Properties

**Figure 3:**
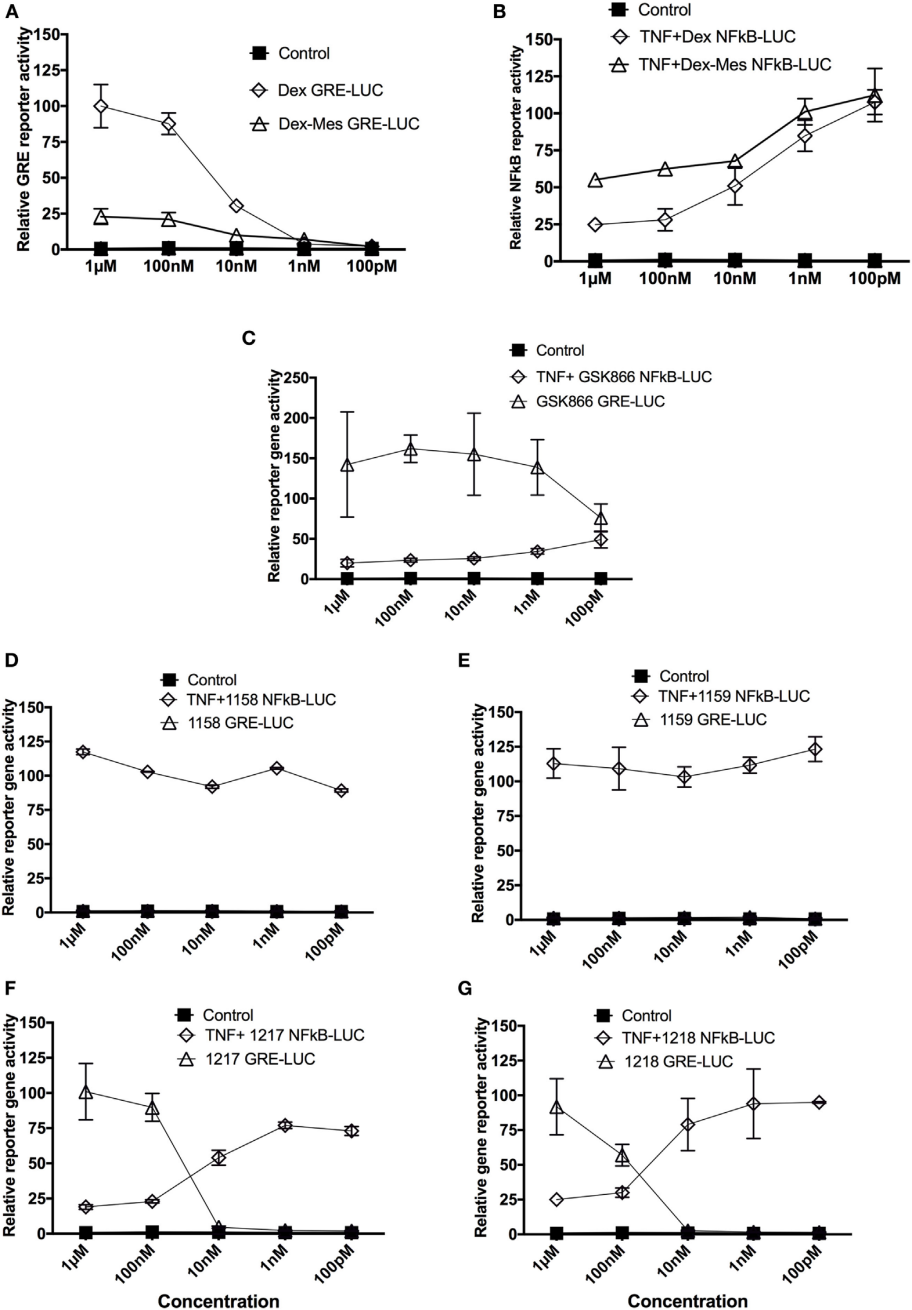
Selective glucocorticoid receptor agonist GSK866 analogs UAMC-1217, UAMC-1218 but not UAMC-1158 and UAMC-1159 elicit selective glucocorticoid receptor-transactivation and NFκB-transrepression properties. Glucocorticoid response element (GRE)- and NFκB-luciferase-dependent reporter gene assays were performed in L929sA and HaCat reporter cells which were stably transfected with, respectively, p(NF**κ**B)_3_-50luc or p(GRE)_2_-50-luc constructs. For p(GRE)_2_-50-luc reporter assays, cells were solvent-treated or induced with different concentrations of Dex **(A)**, Dex-mesylate (Dex-Mes) **(A)**, SEGRA GSK866 **(C)** or analogs UAMC-1158 **(D)**, UAMC-1159 **(E)**, UAMC-1217 **(F)**, and UAMC-1218 **(G)** as indicated for a total exposure time of 6 h, whereas cells were cotreated for TNF (2,500 IU/ml) in the last 4 h for p(NFkB)_3_-50luc reporter assays **(B–G)**. Corresponding lysates were assayed for luciferase activities and normalized for protein content as described in the Section “Materials and Methods.” Promoter activities are expressed as relative induction factor (± standard deviation) i.e., the ratio of expression levels recorded either at induced and non-induced conditions, with the latter taken to be 1.

In a first series of experiments, we compared selective GR-transactivation and NFκB-transrepression effects of the SEGRA GSK866 and potential covalent-binding analogs UAMC-1158, UAMC-1159, UAMC-1217, and UAMC-1218 with non-covalent (Dex) and covalent binding (Dex-Mes) reference GR ligands, *via* GRE- and NFκB-dependent luciferase assays in stable transfected reporter gene cell lines (8, 35). GRE-luc reporter gene cells were exposed for 6 h to the different GSK866 analogs and reference compounds Dex and Dex-Mes in a concentration range of 10^−6^–10^−10^ M (Figure 3). In line with previous reports, Dex-Mes shows weaker dose-dependent GR-dependent transactivation than Dex (Figures 3A,B) (24). Analogous to the covalent GR binding ligand Dex-Mes, adding covalent warheads to GSK866 also significantly reduces the ligand transactivation properties, when compared to the strong GR agonistic activities of GSK866, whereas compounds UAMC-1158 and UAMC-1159 completely fail to elicit any GR transactivation at any dose tested (Figures 3C–E). Interestingly, whereas 10 nM of GSK866 demonstrates significant GR transactivation, similar concentrations of UAMC-1217 and UAMC-1218 show significantly reduced GR transactivation to levels below transactivation observed with 10 nM of Dex (Figures 3F,G). These results correspond with the approx. 5-fold lower GR-LBD affinity for ligands UAMC-1217 (IC50 22 nM), UAMC-1218 (IC50 34 nM) versus GSK866 (IC50 4.6 nM), as demonstrated in ligand displacement assays with radiolabeled Dex (14) (Table 1).

**Table 1:** Cell-based GR ligand-binding competition assay with 1.5 nM [^3^H]-dexamethasone upon competition treatment with 1 µM of the different ligands indicated (14).

Ligand	% inhibition of GR ligand binding	IC_50_ (nM)	K_i_ (nM)
Dex	100		
Dex-mesylate	99	7.3	3.7
GSK866	100	4.6	2.3
1158	−2		
1159	−12		
1217	92	22	11
1218	96	34	17

*% inhibition of GR radioligand binding, IC50 (nM) and Ki (nM) are indicated*.

*Gray shade indicates values not applicable*.

Upon further investigation of ligand-specific anti-inflammatory effects in NFκB-luciferase reporter gene assays, we observed dose-dependent NFκB-transrepression effects of GSK866 and the analogs UAMC-1217 and UAMC-1218, whereas UAMC-1158 and UAMC-1159 are completely ineffective (Figures 3D–G). Interestingly, UAMC-1217 and UAMC-1218 demonstrate stronger anti-inflammatory NFκB repression effects than Dex-Mes and Dex at 10 nM concentration. For example, 10 nM UAMC-1217 and UAMC-1218 both reduce TNF-induced NFκB reporter gene activity approximately 95% (UAMC-1217), 95% (UAMC-1218), whereas Dex-Mes and Dex only show 40–45% inhibition (Figures 3A–F). This, in combination with weaker GR transactivation properties at 10 nM concentrations as compared to Dex, skews the GR towards more selective GR transrepression properties. Similar to the lack of GR transactivation, no transrepression activity could be detected for UAMC-1158 and UAMC-1159 ligands. The latter suggests that UAMC-1158 and UAMC-1159 analogs fail to regulate GR-dependent reporter gene expression, in line with results obtained in cell-based ligand-binding competition assays which failed to show displacement of radiolabeled Dex upon treatment with UAMC-1158 and UAMC-1159 (14) (Table 1).

### SEGRA GSK866 Analog UAMC-1217 Demonstrates Anti-inflammatory IL6 Gene Transcription Concomitantly with Reduced GILZ/TSC22D3 Transcription Levels

**Figure 4:**
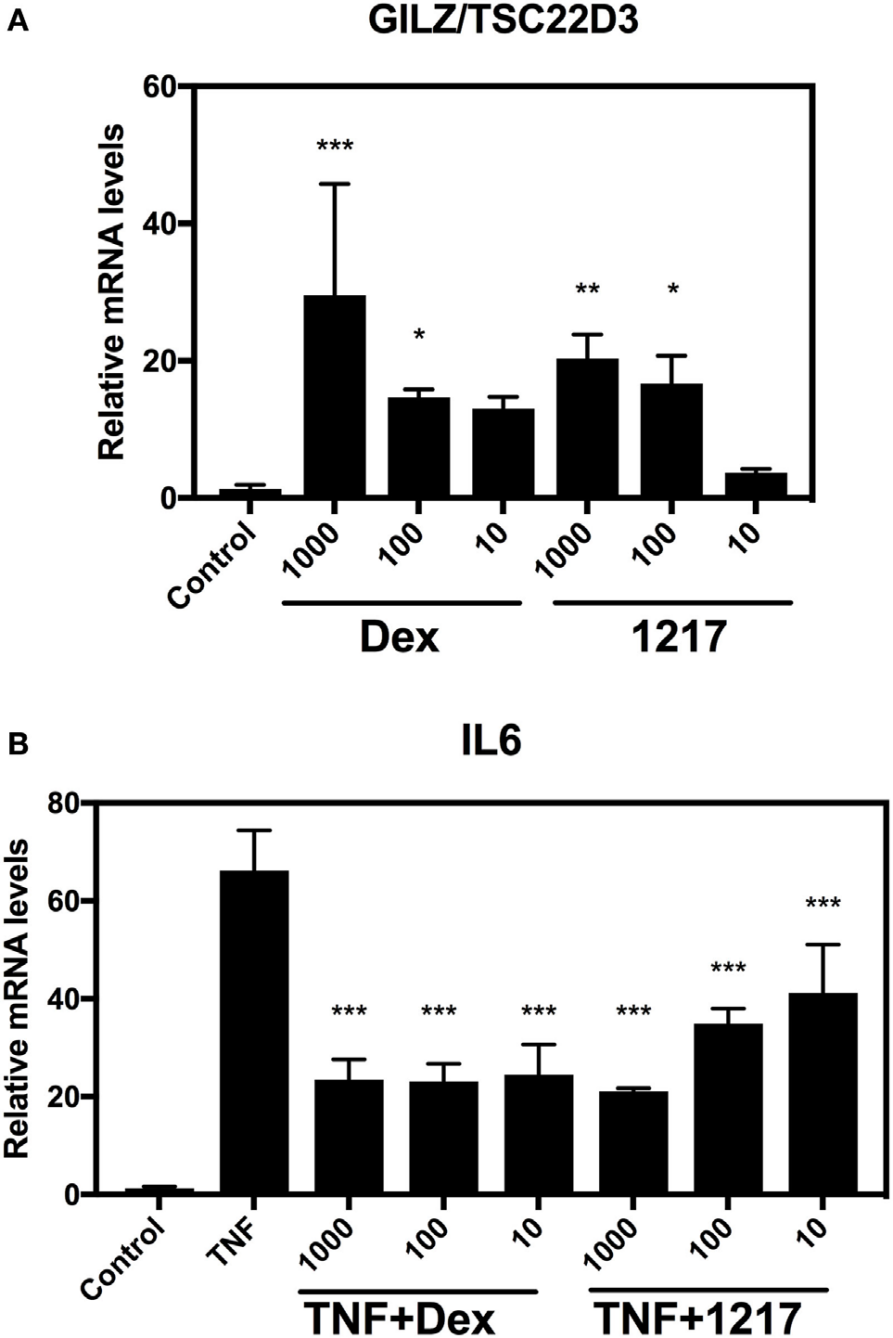
Selective glucocorticoid receptor agonist GSK866 analog UAMC-1217 elicits glucocorticoid receptor selective downstream anti-inflammatory effects at endogenous target genes. **(A)** QPCR assays was performed against the GR target gene GILZ/TSC22D3 in HaCat cells treated for 6 h with different concentrations 10, 100 or 1,000 nM Dex or UAMC-1217. Bar graph plot represents the relative mRNA levels of GILZ/TSC22D3 upon Dex or UAMC-1217 treatment. **(B)** QPCR assays was performed against the TNF-inducible inflammatory gene interleukin-6 (IL6) in HaCat cells treated for 6 h with different concentrations 10, 100, or 1,000 nM Dex or UAMC-1217 either or not, cotreated for the last 4 h with TNF (2,500 IU/ml) as indicated in panel **(B)**. All the qPCR assays have been normalized to the GAPDH mRNA housekeeping gene. Bar graphs represent relative mRNA (mean ± SD) levels of three replicates. Significant mean differences versus control setups panel **(A)** or TNF treated setup panel **(B)** are marked as *** (*P* < 0.001), **(*P* < 0.01), * (*P* < 0.05) and were determined by one-way ANOVA (Dunnetts Posttest).

To confirm GR- and NFκB-specific reporter gene results at the level of endogenous target gene expression in HaCat cells, we next compared effects of 6 h treatment of Dex and UAMC-1217 on mRNA transcription levels of the GR target gene GC-induced leucine zipper (GILZ)/TSC22D3 as well as the inflammatory and TNF-inducible NFκB target gene interleukin 6 (IL6) (Figure 4). In line with the GR- and NFκB-specific reportergene results, we could confirm anti-inflammatory effects of UAMC-1217 in the nanomolar range, with reduced transactivation effects at the level of GILZ/TSC22D3 gene expression.

### SEGRA GSK866 Analogs UAMC-1217, UAMC-1218 Trigger GR Ser211 Phosphorylation

**Figure 5:**
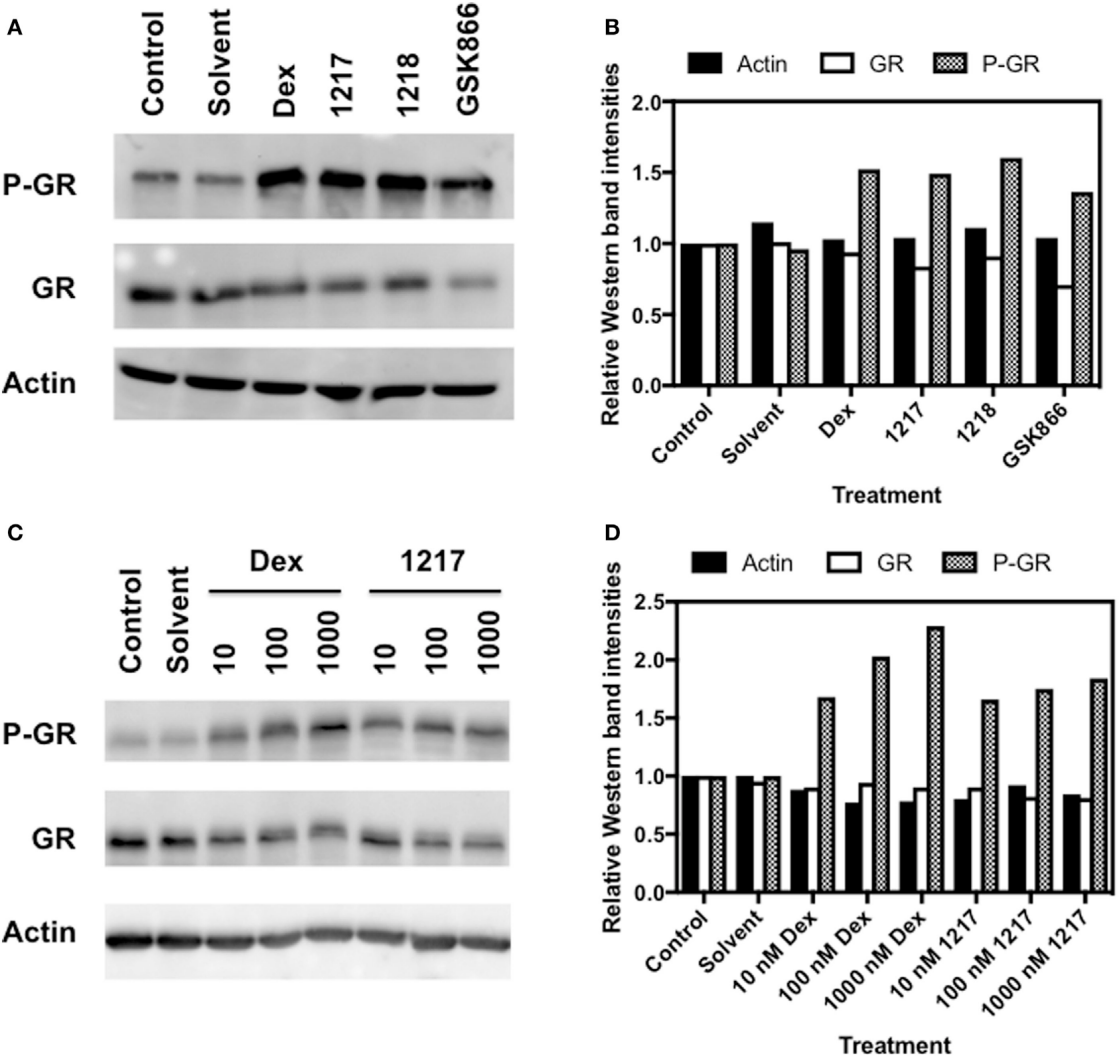
Selective glucocorticoid receptor agonist GSK866 and its analogs UAMC-1217, UAMC-1218 trigger glucocorticoid receptor (GR) Ser211 phosphorylation. **(A)** Western detection of expression levels of Actin, GR, and GR Ser S211 phosphorylation in HaCat cells following 6 h treatment with 1 µM Dex, UAMC-1217, UAMC-1218, or GSK866. **(B)** Bar plot representation of Western blot intensities of Actin, GR, and GR Ser S211 phosphorylation observed in panel A, as quantified by Image J. **(C)** Western detection of expression levels of Actin, GR expression, and Ser S211 phosphorylation in HaCat cells following 6 h treatment with, respectively, 10, 100, or 1,000 nM Dex or UAMC-1217. **(D)** Bar plot representation of Western blot intensities of Actin, GR, and GR Ser S211 phosphorylation observed in panel **(C)**, as quantified by free Image J software.

It is well accepted that hormone-dependent phosphorylation of GR at Ser211 is a marker for transcriptional GR activation (49, 50). Therefore, GR-luciferase reporter cells were exposed for 6 h to Dex, Dex-Mes, GSK866, and SEGRA analogs UAMC-1217 and UAMC-1218 to test their transcriptional GR activation. Total cell lysates were analyzed for expression levels of GR, phosphoGR-Ser211, and actin. From Figures 5A,B, it is clear that SEGRA compound GSK866 and its analogs UAMC-1217 and UAMC-1218 enhance the Ser211 phosphorylation at 6 h, in analogy to Dex. Furthermore, concentration-dependent increase of GR Ser211 phosphorylation can be detected upon Dex or UAMC-1217 treatment (Figures 5C,D). By contrast, the inactive compound UAMC-1158 failed to show any change in GR phosphorylation status upon induction (Figure S2 in Supplementary Material).

### SEGRA GSK866 Analogs UAMC-1217, UAMC-1218, but Not UAMC-1158 and UAMC-1159 Trigger Nuclear GR Translocation

**Figure 6:**
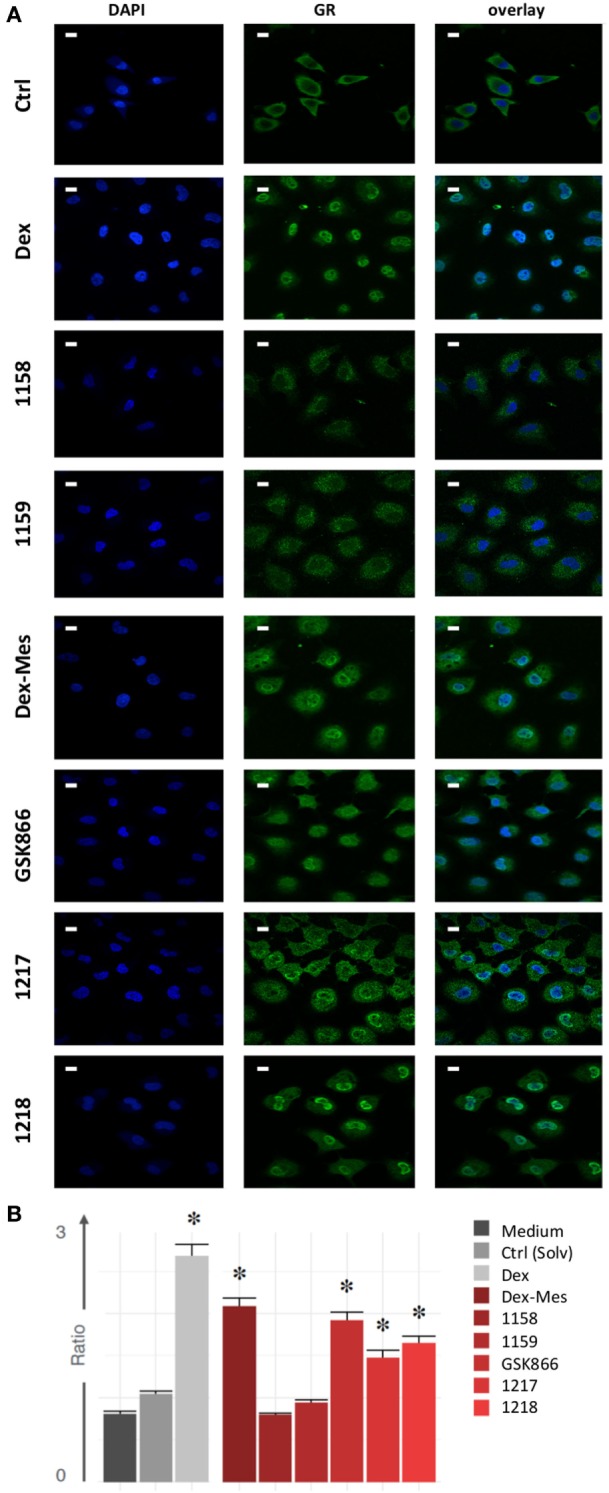
Selective glucocorticoid receptor agonist GSK866 analogs UAMC-1217, UAMC-1218, but not UAMC-1158 and UAMC-1159 trigger nuclear glucocorticoid receptor (GR) translocation. **(A)** Serum starved A549 cells were left solvent treated or treated for 30 min with 1 µM Dex, Dex-mesylate (Dex-Mes), GSK866, UAMC-1158, UAMC-1159, UAMC-1217, or UAMC-1218. GR translocation was evaluated by immunofluorescence whereas nuclei were stained by DAPI dye. Magnification is at 100×. White scalebars represent 20 µM. **(B)** Automated image quantification of GR translocation of three replicate immunofluorescence images of the different experimental setups.

Next, we investigated whether in analogy to Dex and Dex-Mes, SEGRA compound GSK866 and its analogs UAMC-1158, UAMC-1159, UAMC-1217, and UAMC-1218 are able to trigger nuclear translocation of GR in A549 cells, using an immunofluorescence approach, allowing automated image quantification of GR translocation. Serum starved cells were treated for 30 min with mock control (solvent), Dex, Dex-Mes, SEGRA GSK866 and analogs UAMC-1158, UAMC-1159, UAMC-1217, or UAMC-1218. GR translocation was evaluated by indirect immunofluorescence using an anti-hGR antibody as primary antibody and a FITC labeled secondary antibody. Nuclei were stained by DAPI. Nuclear GR translocation was evaluated by overlapping DAPI staining with the GR FITC signal. As can be observed (Figure 6A), besides Dex, also Dex-Mes, GSK866, and its analogs UAMC-1217 and UAMC-1218 induce (partial) translocation of GR from the cytosol to the nucleus, whereas UAMC-1158 and UAMC-1159 are completely ineffective. Similar conclusions can be drawn from the bar graph, which summarizes relative intensities of GR translocation (based on nuclear-to-cytoplasm signal intensity ratio) following standardized image quantification (see Materials and Methods) of the different immunofluorescence pictures (Figure 6B).

### Dex-Mes and SEGRA Compounds UAMC-1217 and UAMC-1218 but Not GSK866 or Dex Bind Covalently to GR-LBD-Specific Cysteines

Next, we further investigated whether the cysteine reactive warheads of the active SEGRA GSK866 analogs UAMC-1217 and UAMC-1218 promote covalent ligand binding to GR-LBD in analogy to Dex-Mes. Ligand-specific interactions with cysteine-containing peptides of GR-LBD were identified by mass spectrometry analysis of trypsin-digested recombinant GR-LBD following incubation with SEGRA compound GSK866, or its analogs UMAC-1217 and UAMC-1218, and reference compounds Dex and Dex-Mes. For all samples, good peptide coverage of the GR-LBD protein could be observed (>85%), and all cysteine-containing peptides were identified following trypsin digestion. Upon screening of all possible trypsin cleaved GR-LBD peptides for mass shifts of the different ligands, covalent binding to GR-LBD could be detected at cysteine C622 for Dex-Mes. Along the same line, covalent binding of GSK866 analogs UAMC-1217 and UAMC-1218 to Cys-622 GR-LBD could also be demonstrated (Table 2). By contrast, no covalent peptide interactions could be demonstrated for Dex, nor the SEGRA GSK866 ligand, as expected, since both lack covalent warheads for covalent interactions. In contrast to previous reports, which identified cysteine 656 as the major target site for covalent binding of Dex-Mes in rat GR, mass spectrometric analysis identified cysteine C622 as the most reactive amino acid for covalent interaction with Dex-Mes in human recombinant GR (51). Of special note, Crystal structure of the human GR-LBD and other human recombinant GR often does not contain the evolutionary conserved Cys655 (corresponding to rat Cys 656), since this amino acid was mutated to glycine to optimize soluble expression and to allow purification of the GR-LBD (52). As such, the current experiments cannot exclude, nor confirm, that Dex-Mes can bind covalently to additional cysteine residues in full-length wild-type hGR in a physiological cellular context. With respect to covalent-binding modes of GSK866 analogs UAMC-1217 and UAMC-1218, mass spectrometry analysis also identified the more solvent exposed cysteine C622 but not the structurally predicted proximal C-643 as the most reactive amino acid for covalent ligand interaction to the GR-LBD. The latter suggests more favorable reaction kinetics for the flexible cysteine C622 than the structural restrained cysteine C643 in covalent ligand binding. Altogether, we confirmed cysteine-dependent covalent binding of Dex-Mes and covalent warhead containing SEGRA compounds UAMC-1217 and UAMC-1218 to GR-LBD.

**Table 2:** Dex-mesylate (Dex-Mes) and selective glucocorticoid receptor agonist (SEGRA) compounds 1217 and 1218 but not GSK866 or Dex covalently bind glucocorticoid receptor ligand-binding domain (GR-LBD)-specific cysteines.

Sample	Modifications
Ctrl	–
Dex	–
Dex-Mes	Peptide 615–633 → C_622_
GSK866	–
1217	Peptide 615–633 → C_622_
1218	Peptide 615–633 → C_622_

*MassspecMass spectrometry-based identification of GR-LBD cysteine-containing peptides which bind covalently to SEGRAs GSK866, 1217, or 1218. The position of the LBD peptide in hGR is indicated by the residue numbers of the first and last amino acid of the peptide (according to the SwissProt database). The modified residue is described by the official amino acid acronym and its position in hGR*.

### Conformational Cofactor Binding Profiling of the GR-LBD Reveals Quantitative Differences in Agonist Conformation by Dex, GSK866, UAMC-1217, and UAMC-1218

**Figure 7:**
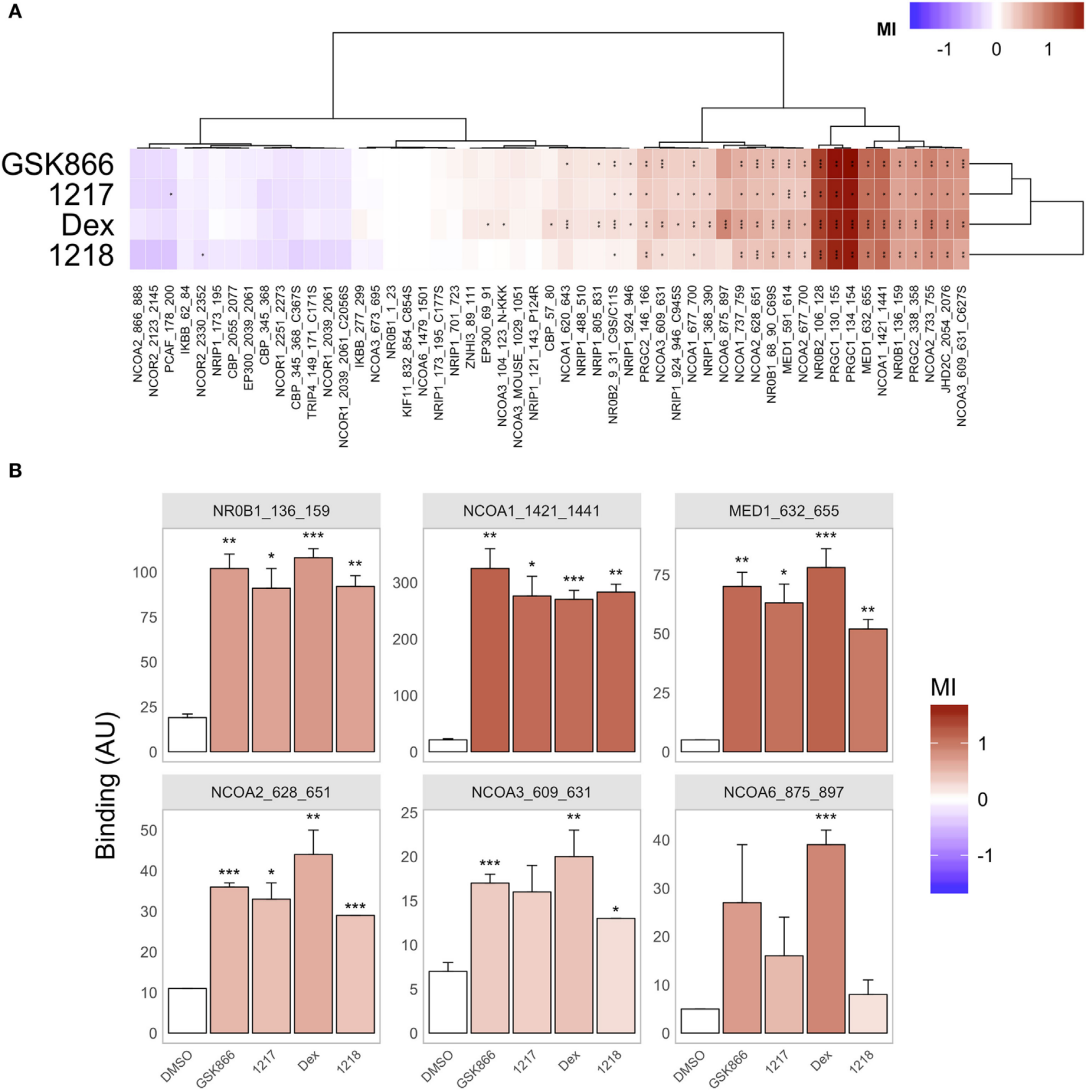
Conformational cofactor binding profiling of the glucocorticoid receptor ligand-binding domain reveals quantitative differences in GR agonist conformation by Dex, or SEGRA compounds GSK866, UAMC-1217, and UAMC-1218. **(A)** Compound-induced log-fold modulation of GR binding [modulation Index (MI)] to coregulator motifs. Red indicates enhancement of binding, while blue represents peptide displacement. (Dis-) similarity between modulation profiles of compounds (rows) and motifs (columns) is visualized by hierarchical clustering (Euclidean distance, Ward’s clustering). **(B)** Bar plot representation of significantly increased coactivator peptide binding profiles. Bar height represents binding (mean ± SD in Arbitrary Units fluorescence) of three technical replicates (arrays) per condition. The color of each bar indicates MI (versus GR binding in the presence of solvent only, DMSO, first bar). Significance of the modulation is indicated by asterisks (*P < 0.05; **P < 0.01; ***P < 0.001).

To further characterize the GR-LBD conformation upon binding of the different active GSK866 analogs, we performed peptide array-based cofactor profiling to fingerprint 53 LxxLL-specific cofactor interactions with recombinant GR-LBD in the presence of the GR agonist Dex, SEGRA GSK866, or the analogs UAMC-1217 and UAMC-1218 (16). By comparing the corresponding peptide interaction profile of the different ligands, to the profiles obtained with Dex agonist bound GR-LBD, it is possible to fingerprint the relative agonistic conformational changes. As can be observed from (Figure 7A), the cofactor profiles of GSK866 and analogs UAMC-1217 and UAMC-1218 promote a similar agonistic cofactor binding profile as Dex treatment. However, whereas some coactivator interactions show similar intensities as Dex (for example, NCOA1_1421_1441), other coactivator interactions (for example, NCOA6_875_897) show significant quantitative differences, suggesting subtle ligand-specific conformational differences underlying its SEGRA properties (Figures 7A,B).

Based on our semi-flexible docking models, we found that spatial occupation of GSK866, and analogs UAMC-1217 or UAMC-1218 in the GR-LBD differ from the binding mode of Dex in the GR-LBD (Figures S3A–E in Supplementary Material). In addition to 4 intermolecular H bonds in the GR-LBD pocket (LEU 563, ASN 564A, GLN 570A, and GLN 642A), GSK866 also forms non-H bond interactions (MET 560A, PHN 623A, GLN 642, and TYR 735) (Figure S3A in Supplementary Material). Upon molecular docking of UAMC-1217 and UAMC-1218 in the GSK866 bound GR-LBD crystal structure (3E7C), stable binding energies of −10.6 and −10.3 kcal/mol were obtained, respectively. The pyrazole group and dichlorophenyl ring of UAMC-1217 forms π bonds with GLN 642 and MET-604, whereas MET604, PHE623, ASN564, LEU563, LEU608, and GLN642 residues constitute strong hydrophobic interactions (Figure S3C in Supplementary Material). Hence, as expected, the interaction profile of UAMC-1217 (Figure S3C in Supplementary Material), more closely resembles GSK866 (Figure S3A in Supplementary Material) than Dex binding to GR-LBD (Figure S3E in Supplementary Material).

### Molecular Dynamic Simulations of GR-LBD Bound to the Novel SEGRA Compound UAMC-1217

**Figure 8:**
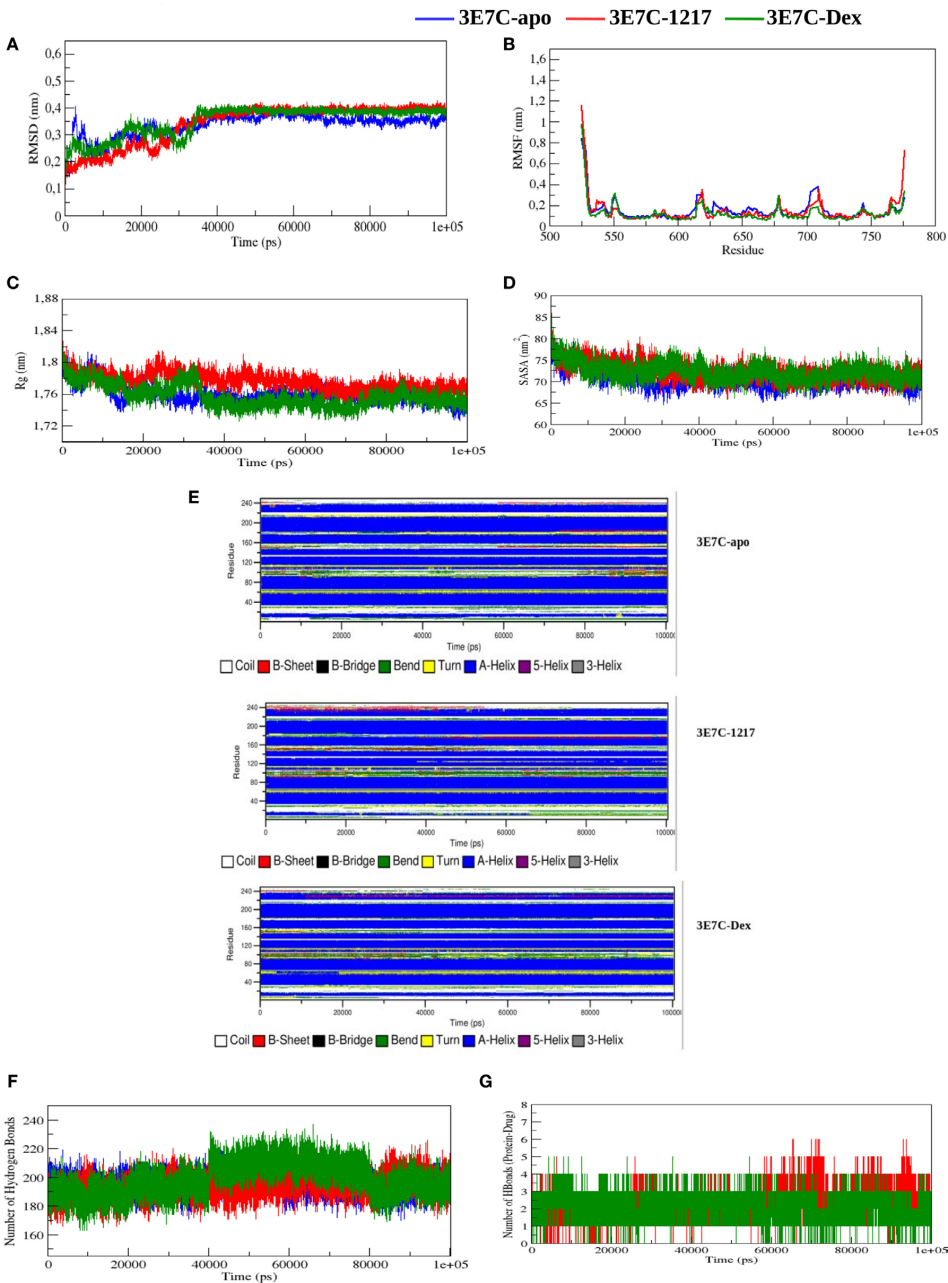
Molecular dynamic simulations of glucocorticoid receptor ligand-binding domain bound to the novel SEGRA compound UAMC-1217. **(A)** RMSD of 3E7C-apo, 3E7C-1217, and 3E7C-Dex versus time at 300 K. **(B)** RMSF of the backbone of C-alpha. **(C)** Radius of gyration of C-alpha atoms. **(D)** Solvent-accessible surface area of 3E7C-apo, 3E7C-1217, and 3E7C-Dex versus time at 300 K. **(E)** Time evolution of the secondary structural elements of 3E7C-apo, 3E7C-1217, and 3E7C-Dex at 300 K (DSSP classification). **(F)** NH-bond of 3E7C-apo, 3E7C-1217, and 3E7C-Dex versus time at 300 K. **(G)** Average number of intramolecular hydrogen bonds between protein and drug.

To predict transient dynamic changes in GR-LBD structure upon binding to an active SEGRA analog UAMC-1217 we performed molecular dynamic (MD) simulations. Due to computational time limitations, the MD simulations could not be repeated for all ligands included in our study. As input for the MD simulations we selected the structural model of SEGRA compound UAMC-1217, bound to the GR from the crystallized 3E7C protein-ligand complex, with an optimized binding energy of −10.6 kcal/mol (Figure S3 in Supplementary Material). In order to compare dynamic conformational variations of the GR-LBD (3E7C) upon interaction with SEGRA UAMC-1217, we studied the root mean square deviation (RMSD), root mean square fluctuation (RMSF), radius of gyration (Rg), solvent accessible surface area (SASA), number of hydrogen bonds (H-bonds), and secondary structure analysis (DSSP) between the apo GR-LBD (3E7C-Apo) form and GR-LBD form bound to UAMC-1217 (3E7C-1217) or Dex (3E7C-Dex) ligand. From the start to ~35,000 ps, the RMSD of the backbone atoms of the 3E7C–1217 complex structure shows significant differences as compared to 3E7C-apo, during the simulation (Figure 8A). After the time period of ~35,000,000 ps, the RMSD of both complex structures (3E7C-1217 and 3E7C-Dex) and 3E7C-apo show a similar profile till the end of the simulation. It is clear that the RMSD of the backbone atoms of both the 3E7C-1217 and 3E7C-Dex complex and 3E7C-apo reach a stable conformation. The average RMSD values of the 3E7C (apo), 3E7C-1217 and 3E7C-Dex complex structures are presented in Table 3.

**Table 3:** Average values of root mean square deviation (RMSD), root mean square fluctuation (RMSF), radius of gyration (Rg), solvent accessible surface area (SASA), number of hydrogen bonds (NH-bonds), and NH-bonds between protein and Drug of 3E7C-apo, 3E7C-UAMC-1217 and 3E7C-Dex.

Parameters	3E7C-Apo	3E7C-1217	3E7C-Dex
RMSD (nm)	0.34 ± 0.04	0.34 ± 0.07	0.35 ± 0.05
RMSF (nm)	0.15 ± 0.10	0.14 ± 0.13	0.11 ± 0.10
Rg (nm)	1.76 ± 0.01	1.78 ± 0.01	1.76 ± 0.01
SASA (nm^2^)	70.54 ± 2.15	72.23 ± 2.16	72.25 ± 2.04
NH-bonds	194.86 ± 6.99	194.61 ± 7.33	198.67 ± 11.05
NH-bonds (protein-drug)	–	2.06 ± 0.80	1.95 ± 0.67

To analyze the flexibility behavior of the 3E7C protein upon binding of UAMC-1217 and Dex, we calculated the RMSF of Cα atoms of each residue. In Figure 8B, a minor fluctuation can be observed in 610–660 region (helix 5) and 700–720 region (loop region 11), which indicates that UAMC-1217 and Dex binding to the GR-LBD complex elicit subtle differential effects in the molecular dynamics as compared to the Apo form. Furthermore, the average RMSF values of the both complex structures are somewhat lower than 3E7C-Apo structure (Table 3). This is further supported by the surface and geometry analysis of 3E7C upon binding of UAMC-1217 or Dex, by analyzing the Rg and SASA (Figures 8C,D). In both the Rg and SASA plot, the 3E7C-1217 complex shows a slightly larger structural deviation between ~16,000 and 60,000 ps than the 3E7C-Apo and 3E7C-Dex structures. Overall, the average values of Rg and SASA (Table 3) show a comparable binding pattern for UAMC-1217 and Dex GR-LBD complex structures. To understand the pattern recognition of hydrogen-bonded and geometrical features in the secondary structures before and after the complex, we used NH-bond analysis and the DSSP program (53). The most significant changes in the residue position of 600–630 (indicated as 75–110 in Figure 8E) and 700–720 (indicated as 174–194 in Figure 8E) show a higher helical content in the both 3E7C-1217 and the 3E7C-Dex complex structures. Altogether, these results suggest that the docked complex structures (3E7C-1217 and 3E7C-Dex) are stable over time. To confirm this, we performed intermolecular NH-bond analysis. Due to the helical content, the ligand bond GR-LBD structures show more H-bonds than the 3E7C-apo structure (Figure 8F). Furthermore, we also observed intramolecular hydrogen bonds between the protein and drug during the simulation time period (Figure 8G). It clearly demonstrates that the 3E7C-1217 complex shows very similar ligand-binding dynamics as the 3E7C-Dex complex. Besides, the 3E7C-1217 complex shows a slightly higher number of intramolecular H-bonds than the 3E7C-Dex complex. Overall, MD simulation analysis collectively suggest that, besides subtle transient dynamic changes, average GR-LBD conformations upon binding to UAMC-1217 or Dex show a similar way of ligand-binding dynamics which leads to a stable interaction between the protein and ligand molecule.

## Discussion

Topical GCs are the most commonly prescribed anti-inflammatory agents in management of many cutaneous inflammatory diseases and the preferred mode of application is by topical administration of a corticoid agonist. However, long-term use of GCs is associated with many side effects such as skin atrophy, systemic toxicity, and rebound effects after discontinuation of the treatment. Current therapeutic regimens available at the market for topical applications include steroid analogs, such as clobetasol, mometasone (Elocom), and bethamethasone. Meanwhile, also non-steroidal SEGRAcompounds have been designed with more selective anti-inflammatory properties and less adverse side effects, such as CpdA (38), ZK245186 (54), and GSK866 (15). The aim of the current study was to evaluate potential SEGRA properties of novel GSK866 analogs with covalent warheads for more selective anti-inflammatory properties. The reactive anchor group in the ligand acts as an electron-deficient Michael acceptor to target nucleophilic thiol rich cysteines in the ligand-binding domain of GR. Such an approach is conceptually attractive, since having a long-lasting covalent linkage between the ligand and the GR might potentially prolong the therapeutic effect and reduce adverse systemic side effects, since smaller doses of the drug can be applied. Novel SEGRA GSK866 analogs with potential cysteine C643 reactive covalent warheads were designed *in silico* and synthesized (14) for further molecular characterization of SEGRA properties in cell-based assays.

Dose–response studies of the different GSK866 analogs in GRE- and NFκB-dependent reporter gene studies revealed consistent anti-inflammatory but reduced GR transactivation effects for GSK866 analogs UAMC-1217 and UAMC-1218 at nM concentrations, whereas analogs UAMC-1158 and UAMC-1159 were found to be completely inactive at μM concentrations. Similar results were obtained at the endogenous gene level, which revealed dose-dependent immunosuppressive effects of UAMC-1217 and Dex, which dose-dependently reverse TNF induced IL6 gene expression, whereas maximal GR-dependent GILZ/TSC22D3 gene transcription is reduced for UAMC-1217 in comparison to Dex treatment.

Along the same line, GR Ser211 phosphorylation and nuclear GR translocation could be observed upon cellular treatment with GSK866 analogs UAMC-1217 and UAMC-1218, whereas no effects could be observed upon treatment with UAMC-1158 or UAMC-1159. These results are in line with [^3^H]-dexamethasone radioligand binding competition assays (Table 1) (14), which show near complete displacement of GR ligand binding with the SEGRA GSK866, and synthetic analogs UAMC-1217 and UAMC-1218, whereas compounds UAMC-1158 and UAMC-1159 are completely inactive. As such, it cannot be excluded that cellular metabolization of UAMC-1158 or UAMC-1159 compounds or their promiscuous reactivity may prevent GR specific ligand activity.

Furthermore, SEGRA properties of UAMC-1217 and UAMC-1218 were further supported by conformational cofactor binding profiling experiments of recombinant GR-LBD with the MARCoNI peptide array (16, 55). Whereas most coactivator interactions mimicked ligand-binding effects of the full agonist Dex, we observed a subtle conformational difference of the GR-LBD with a few coactivator peptides, which may finetune SEGRA properties. However, one limitation of our approach is that only ligand induced allosteric GR-LBD conformational changes are evaluated in the cofactor profiling, whereas additional GR-LBD-independent or allosteric control mechanisms induced by the DNA-responsive element are not taken into account, which may also contribute in DNA-context-dependent SEGRA properties (56).

Finally, molecular dynamic (MD) simulations were performed to detect transient dynamic changes in GR-LBD structure upon binding to UAMC-1217 as compared to a Dex liganded or unliganded (Apo) GR-LBD E7C crystal structure. Due to computational limitations, molecular dynamic simulations were restricted to compound UAMC-1217 which was predicted to be the SEGRA GSK866 analog with the most stable GR-LBD binding energy (−10.5 kcal/mol, according to Autodock Vina). Interestingly, RMSD and RMSF simulations revealed transient (from start to ~35,000 ps) conformational differences with Dex binding to GR-LBD across helix 5 at positions 600–630, including the cysteine C622 which was identified as a covalent target for 1217 in mass spectrometric analysis. Later than 35,000 ps, simulation predicts disappearance of the conformational differences and both 1217 or Dex complexed GR-LBD conformations evolve toward similar stable structures. Remarkably, although molecular modeling identified cysteines C643 and C736 in the GR-LBD as the most favorable proximal targets for covalent binding of SEGRA compounds UAMC-1217 and UAMC-1218, mass spectrometry analysis identified the solvent exposed C622 as the most reactive site for covalent binding by UAMC-1217, UAMC-1218, and Dex-Mes. However, whether 1217 targets additional cysteine residues present in full-length GR besides GR-LBD C622 needs further investigation.

In conclusion, by combining various molecular cellular assays, we demonstrate that GSK866 analogs UAMC-1217 and UAMC-1218 hold promise as a novel class of covalent-binding SEGRA ligands for treatment of inflammatory disorders. Development of therapeutic SEGRA agonists with stronger anti-inflammatory potential with less side effects in cutaneous and ocular inflammatory diseases is promising area for therapeutic intervention (57–62). In this respect, future investigations will be required to translate preclinical *in vitro* results of covalent SEGRA compounds UAMC-1217 and UAMC-1218 into clinical (topical) applications for treatment of long-lasting skin and/or ocular inflammatory disorders.

## Author Contributions

Conceptualization: CSC, AP, JJ, PVDV, KA, XVO, AB, HW, and WVB. Wet lab experiments: CSC, AP, KD, MWCV, BR, RO, KDB, NB. Data analysis: CSC, AP, BK, WHDV, RH, HDW, and WVB. Supervision: KDB, KG, PV, KA, XVO, AB, HDW and WVB. Writing: CSC, AP, WHDV, BR, KDB, KG, XVO, AB, HDW and WVB. Funding: KA, XVO, and WVB.

## Conflict of Interest Statement

The funders had no role in study design, data collection and analysis, decision to publish, or preparation of the manuscript. The authors declare that they have no competing financial interests.

## Supplementary Material

Figure S1 Automated image analysis of nuclear glucocorticoid receptor (GR) translocation by determining the ratio nuclear region of interest versus cytoplasmic region of interest (see Materials and Methods).

Figure S2 **(A)** Western detection of glucocorticoid receptor (GR) expression and Ser S211 phosphorylation levels in p(GRE)_2_-50-luc reporter Hacat cells following 4 or 24 h treatment with 1 µM Dex, GSK866, UAMC-1158, UAMC-1217, or UAMC-1218. **(B)** Western detection of GR expression and Ser211 phosphorylation levels in p(GRE)_2_-50-luc reporter cells following 4 h solvent treatment, or exposure to 1 µM Dex, Dex-mesylate (Dex-Mes), or UAMC-1217.

Figure S3 Molecular 2D–3D modeling of selective glucocorticoid receptor agonist GSK866 **(A)** and synthetic analogs UAMC-1158 **(B)**, UAMC-1159 **(C)**, UAMC-1217 **(D)**, UAMC-1218 **(E)** in the glucocorticoid receptor ligand-binding domain crystal structure 3E7C.
