# Supplementary figures and images for: Selective Glucocorticoid Receptor Properties of GSK866 Analogs with Cysteine Reactive Warheads
Source: Front Immunol. 2017 Nov 1;8:1324. doi: 10.3389/fimmu.2017.01324 (PMC5672024; doi:10.3389/fimmu.2017.01324)

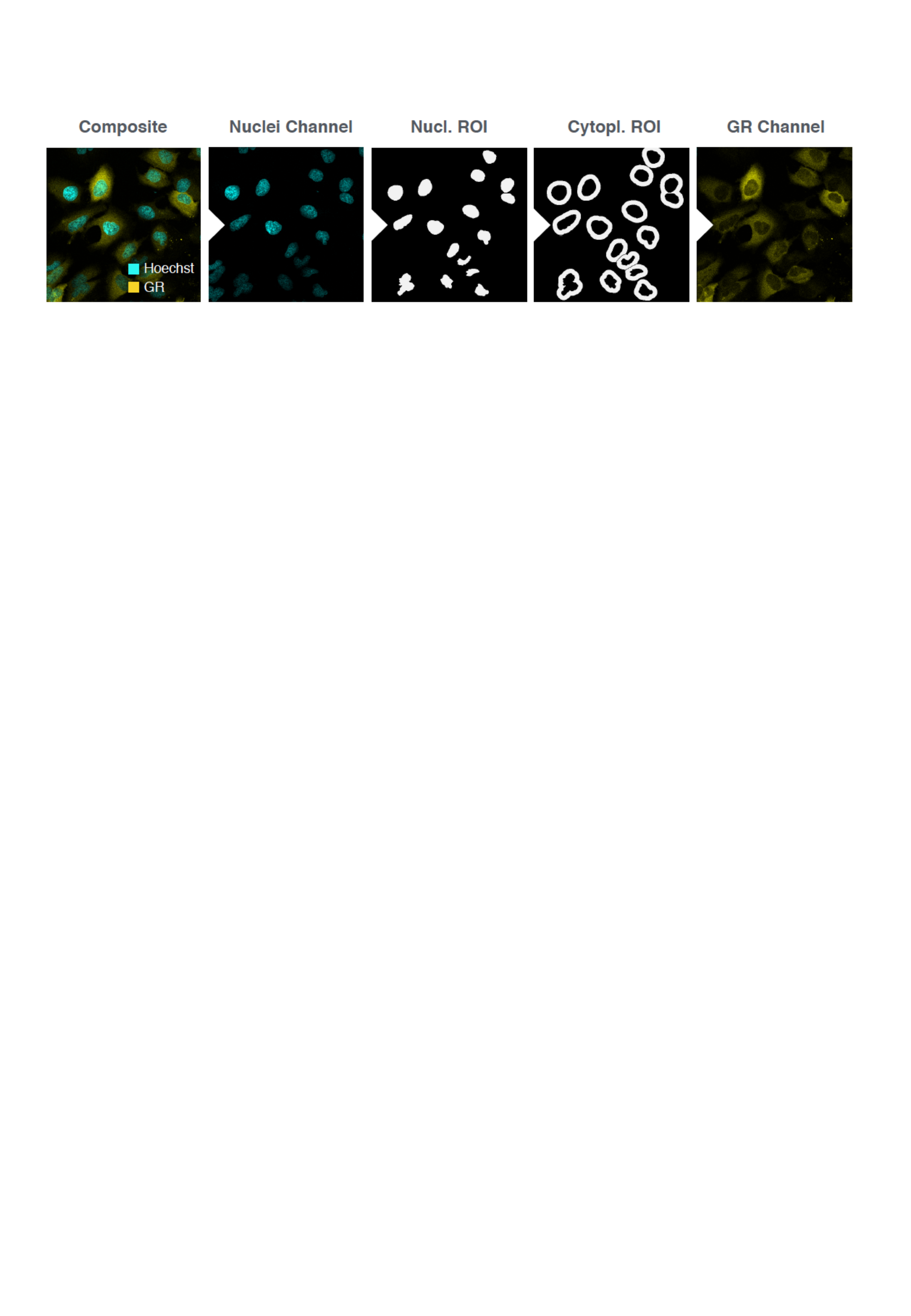

Supplement: Figure S1 — Automated image analysis of nuclear glucocorticoid receptor (GR) translocation by determining the ratio nuclear region of interest versus cytoplasmic region of interest (see Materials and Methods). [file image_1.tiff]

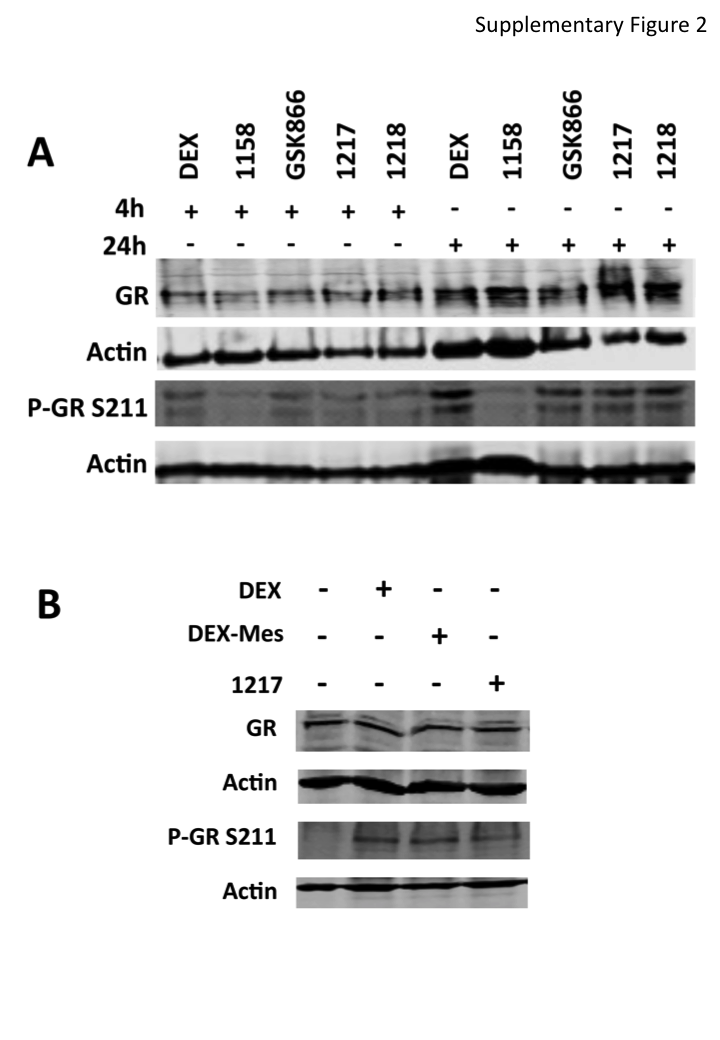

Supplement: Figure S2 — (A) Western detection of glucocorticoid receptor (GR) expression and Ser S211 phosphorylation levels in p(GRE)2-50-luc reporter Hacat cells following 4 or 24 h treatment with 1 µM Dex, GSK866, UAMC-1158, UAMC-1217, or UAMC-1218. (B) Western detection of GR expression and Ser211 phosphorylation levels in p(GRE)2-50-luc reporter cells following 4 h solvent treatment, or exposure to 1 µM Dex, Dex-mesylate (Dex-Mes), or UAMC-1217. [file image_2.tiff]

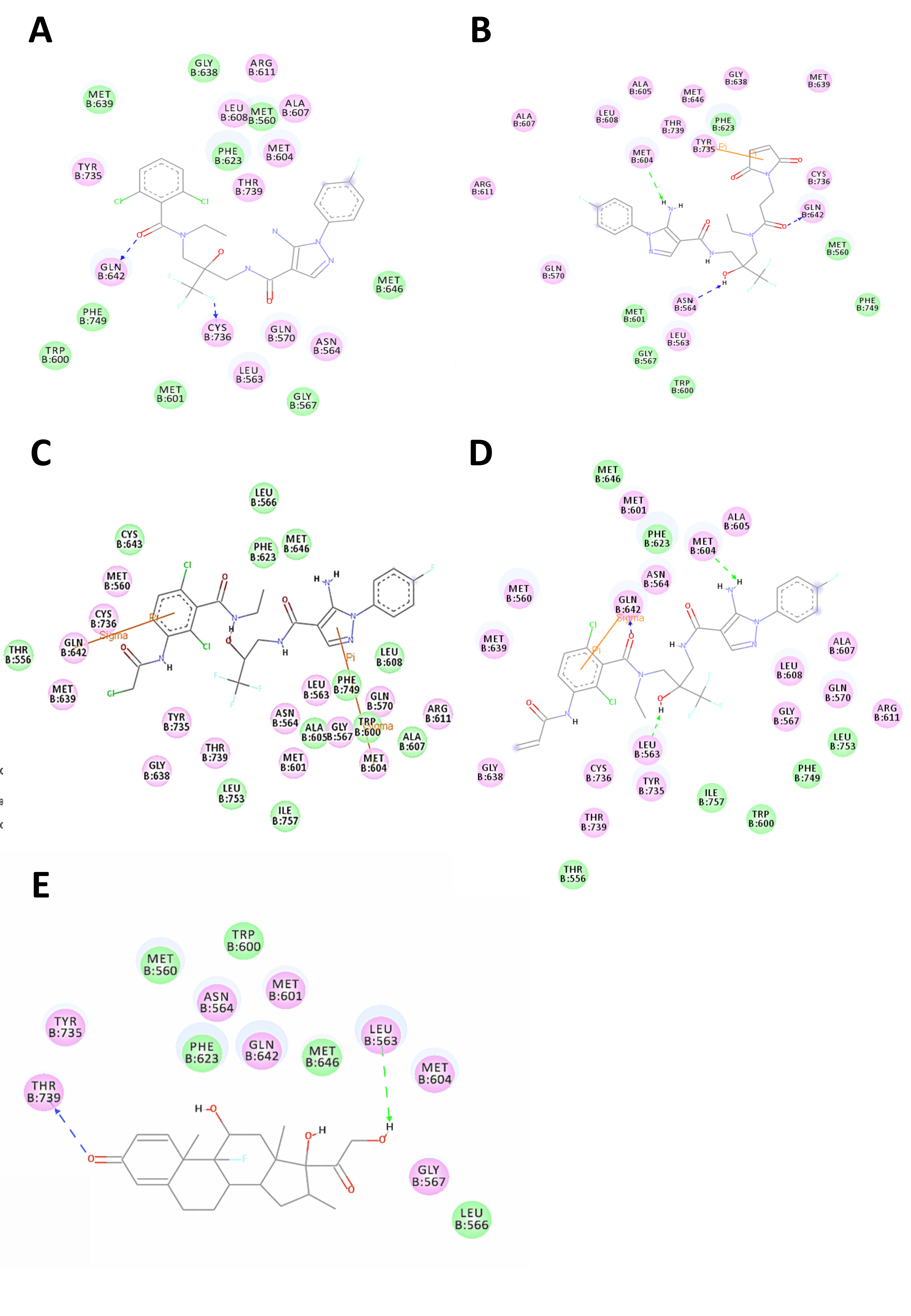

Supplement: Figure S3 — Molecular 2D–3D modeling of selective glucocorticoid receptor agonist GSK866 (A) and synthetic analogs UAMC-1158 (B), UAMC-1159 (C), UAMC-1217 (D), UAMC-1218 (E) in the glucocorticoid receptor ligand-binding domain crystal structure 3E7C. [file image_3.tiff]
